# Data processing techniques to improve data integration from dairy farms

**DOI:** 10.3168/jdsc.2024-0723

**Published:** 2025-03-12

**Authors:** Jacquelyn P. Boerman, Luiz F. Brito, Maria E. Montes, Jacob M. Maskal, Jarrod Doucette, Kirby Kalbaugh

**Affiliations:** 1 Department of Animal Sciences, Purdue University, West Lafayette, IN 47907; 2 Agriculture Data Services, Purdue University, West Lafayette, IN 47907

## Abstract

Large-scale data generation on dairy cattle farms is expected to continue increasing due to more animals per farm and the adoption of on-farm sensors and technologies that generate additional information on individual animals in greater frequency. Siloed data and information, lacking interoperability, prevent end users from combining data from multiple data sources and drawing more meaningful conclusions from the data generated on farm. As a result of these data challenges, the objective of this technical note is to describe a process of designing and documenting the development of a data ecosystem that automatically collects, performs quality control, and integrates data from disparate data sources used on experimental and commercial dairy farms. Integrated data can be queried to answer specific questions or generate timed reports that provide more insight than singular data sources can provide. Our objective was to develop a collaborative research data infrastructure that enables comprehensive data accessibility through an integrated computational ecosystem comprising open-source technologies of JupyterHub, Python, and Apache Spark. This shared curated environment facilitates extensive dataset consumption, empowering users to leverage distributed computing resources and parallel processing capabilities for sophisticated multi-dataset analysis and integration. Before user accessibility, the farm data undergo a rigorous multistage preprocessing protocol designed to mitigate potential data integrity challenges. These comprehensive data curation steps systematically address complex variability with sources, including vendor-specific software modifications, intermittent data retrieval disruptions, and farm-level operational contingencies. Employing sophisticated data cleaning, transformation, and validation methodologies, the infrastructure ensures robust data standardization and quality assurance. The integration of datasets from different data sources is paramount for improving dairy cattle welfare and production efficiency, which are complex management and breeding goals influenced by a multitude of traits that can be measured by different sensors. We identified research and further development needed in the field of dairy data science (e.g., data editing and quality control procedures, references and standards for novel sensor-based variables, and validation of obtained data across sensors), which is expected to continue playing a major role in the dairy industry sustainability.

## Highlights

• The described data ecosystem integrates multiple dairy farm-derived data sources.
• Standardized data processing techniques ensure data integrity.
• Using multiple data tools allows for efficient data processing and analysis.

## Graphical Abstract

**Figure:**
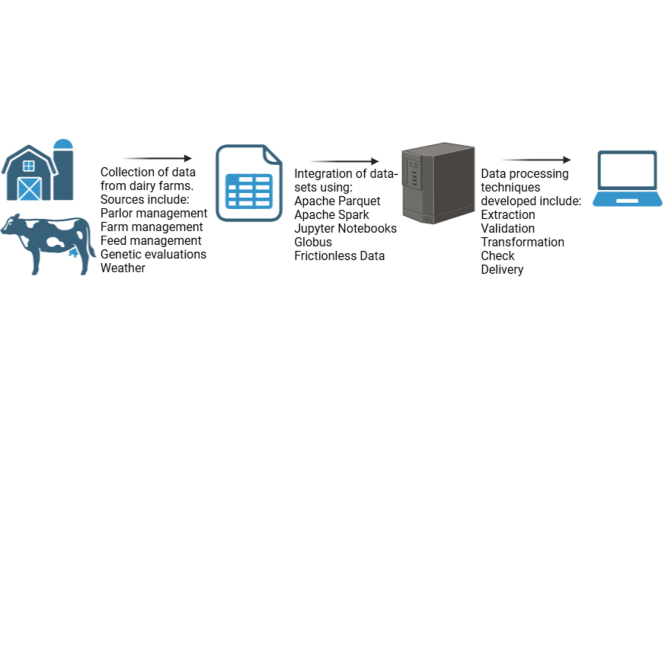
**Summary:** Dairy producers are using multiple software programs and sensors on their farms to assist with farm management and generating reference datasets for breeding purposes. A key challenge to optimize the use of all data generated on dairy farms is the lack of data operability across data sources and limited data integration. In this context, we have developed the Purdue Animal Science Data Ecosystem (PASDE) to facilitate the integration of various dairy data sources. In this technical note, we describe the data processing techniques used for data ingestion, quality control, and data integration to allow for complex biological processes to be explored. In addition, we provide examples of studies developed using the integrated datasets.

The development of precision livestock farming (**PLF**) has increased the phenomenon of electronic information transfer to measure and model data collected in animal production systems. The goal of collecting this information is to optimize both farm-level and individual animal management, and to develop novel traits for the purposes of breeding and improve selection schemes. The increase in available data without proper integration has resulted in data becoming too cumbersome for producers to use, running the risk of this resource having little impact on daily farm management. The use of sensors on dairy farms is widespread. For example, Stygar et al. (2021) identified 129 different technologies with applications for animal-based welfare assessment, including accelerometers, cameras, milk sensors, boluses, and load cells. However, only 18 of the 129 animal welfare technologies were externally validated. To ensure that the value of these new PLF sensors and technologies is realized and efficiently integrated with routinely collected data, there is an urgent need to develop and implement best practices for handling the data generated from them and their integration with existing technologies and traditional data recording systems.

The data generated from multiple sensors and management software programs have reduced utilization if these programs fail to communicate and interact with each other. Data interoperability is the ability to share and make use of data between devices and systems using a common infrastructure (López-Morales et al., 2020). The ruminant farm system (**RuFaS**) model is an example of the need for interoperable data as it relies on inputs from multiple areas (i.e., animal, manure handling, crop and soil, and feed storage to run whole farm simulations; Hansen et al., 2021). As researchers explore more complex relationships requiring data from multiple sources, interoperability becomes critical. Data interoperability issues, including spatiotemporal differences between data sources, different data structures and formats, lack of common data identifiers (i.e., non-unique animal ID), and different language frameworks used, prevent the full usage of data generated on farms. Therefore, there is a need for developing data infrastructure for integrating information collected from multiple data sources to understand the complex relationships on dairy farms between the animals, environment, and inputs and outputs of the system. We are proposing methods to generate a data ecosystem, with a focus on dairy farm data, that includes a series of steps for best management practices for the collection, editing, quality control, and integration of data across data sources.

The ingestion of data is the physical transfer of data from the source into a data ecosystem and it can be accomplished by specifically designed software or using application programming interfaces (**API**) to retrieve and transform data (Sawadogo and Darmont, 2021). Integration of multiple data types has increased our understanding of the complex relationships between individual animals, populations of animals, and their environment. Although the benefits of data integration may seem obvious, the methods for performing data integration across data types remain underdeveloped with many challenges to be addressed. Therefore, the primary objective of this technical note is to describe a process of designing and documenting the development of a data ecosystem that automatically collects, performs quality control, organizes, and integrates data from disparate data sources used on commercial dairy farms. This data ecosystem can then be queried to answer specific questions with timed reports generated as well as the development of application tools and research projects.

The data ecosystem, termed Purdue Animal Science Data Ecosystem (**PASDE**), has been developed to integrate various data sources. The datasets used were generated in commercial and experimental dairy farms located in the state of Indiana (USA). These farms use different software programs and sensors. The PASDE automatically gathers and integrates information from a variety of sources. Examples and details of the data sources are listed here:

1 Parlor management (AfiMilk, Kibbutz Afikim, Israel) is a sensor and software system that records per animal activity and milking information. An AfiTag is placed on dairy cows and these AfiTags record information about cow activity and milking information with data captured during each milking events daily. The data are collected, and reports are autogenerated nightly to represent data from the previous 24 h.
2 Farm management: PCDart (Dairy Record Management Systems, Raleigh, NC) and DairyComp 305 (Valley Ag Software, Visalia, CA) are herd management software systems that record information about individual animals including their identification, health events, EBV, milk production variables, and reproduction records. Data are entered into the system in a variety of ways including manual recording of events or importing information after a test-day when milk samples were taken from individual cows.
3 A genetic evaluation system (e.g., Zoetis, Parsippany, NJ) provides genomic information for individual animals. This information includes thousands (∼50,000) of SNP per genotyped animal together with regular genomic prediction of breeding values for dozens of traits and the respective metadata in the form of “.csv” files.
4 Weather information (e.g., Synoptic Data, Fairfax, VA) is the source of weather information from a local weather station located close to the dairy farms. The weather data includes the location of the weather station, temperature, precipitation, cloud cover, and wind speed. Data are recorded every 15 min. Weather station data are obtained daily through Synoptic Data (Mesonet network) through their API and saved in the form of a “.csv” file.
5 Feed management: TMR Tracker (Digi-Star, LLC, Fort Atkinson, WI) is a feed management software program that records information about ingredients used, time mixed, and weights of diets mixed. Data are recorded onto a local software system and multiple reports are run daily and saved as “.csv” files.
6 Automated milking systems (milking robots): Lely (Maassluis, the Netherlands) is a milking technology that gathers per milking visit, quarter-specific records from individual cows. Records contain information on milk production, milk composition, cow health monitoring (e.g., milk temperature and milk electrical conductivity), milking duration, and udder characteristics. Additionally, Lely Qwes (Maasslius, the Netherlands) technology allows for collection of measures of activity and rumination.
7 Automated milk feeders provide data on milk consumption for individual calves, including data per visit and number of visits to the milk feeder per day.

The integration of all these datasets requires stacking of various technologies. The chosen technology stack makes the PASDE system both scalable and domain agnostic. While not necessary to make the system operational, the stack is able to be containerized, thus creating a template that can be deployed on a wide vary of computing platforms and at a variety of scales. The system also is not domain specific to dairy farming, and thus could be used for any large tabular datasets. The components include

1 Apache Parquet (https://parquet.apache.org/) is ideal for PLF research due to its highly efficient columnar storage design. Files also contain metadata about the data in each file. Column-level metadata like data types, minimum and maximum values, and counts of null values make data analysis faster than row-based file storage. Its support for complex nested data structures makes it particularly valuable for genomics, animal activity, climate, and large data analytics, where users often work with multidimensional datasets. The format's compatibility with distributed computing frameworks such as Apache Spark, with its built-in compression algorithms like Snappy and gzip, can reduce file sizes by up to 75% without affecting data integrity.
2 Apache Spark (https://spark.apache.org/): Spark is excellent for data cleaning, processing, and transforming. Spark supports multiple programming languages, including Python, Scala, Java, and R, providing users flexibility in their workflows. Its comprehensive libraries enable sophisticated data manipulation and analysis methods, including Spark SQL for structured data, MLlib for machine learning, GraphX for network analysis, and Spark Streaming for real-time data processing. Spark's resilient distributed datasets and dataframe abstractions allow users to perform parallel computations across large computer clusters or even on individual workstations. Spark's fault-tolerance mechanisms can automatically recover from node failures during distributed computing tasks, increasing reliability.
3 Jupyter Notebook (https://jupyter.org/) is platform-independent and can be accessed via web browsers. It supports version control systems, making it suitable for collaborative projects, where multiple team members can work on the same notebook simultaneously. One of the key strengths of Jupyter Notebook is its seamless integration of programming code, text, and visual elements within a single document. Users can create and run code cells, which allow for live code execution, making it ideal for iterative data analyses and experimentation. This live execution capability enables users to see the immediate results of the code, making it easier to debug and refine data analysis processes.
4 Globus (https://www.globus.org/) is a research data cyberinfrastructure, developed and operated as a not-for-profit service by the University of Chicago (Chicago, IL). It is a reliable method for transferring and sharing both large and small amounts of data. Local clients installed on endpoints as well as research data infrastructure can be copied, moved, and shared using a web interface or scripts to automate processes.
5 Frictionless Data (https://frictionlessdata.io) provides a comprehensive, open-source toolkit that addresses critical challenges in data management and interoperability. The framework provides standardized, machine-readable (JSON or YAML) metadata descriptions of datasets through its Data Package specification (https://specs.frictionlessdata.io/), which ensures comprehensive documentation, validation, and portability of research data. Frictionless has cross-platform compatibility and support for multiple programming languages and data formats to facilitate seamless data integration. The framework's emphasis on open standards and interoperability aligns perfectly with the emerging global push for open science, enabling users to create FAIR (findable, accessible, interoperable, and reusable) datasets.

The steps of automated data integration are extraction, validation, transformation, checking, and delivery as shown in Figure 1. The data do not move to the next step at each process stage unless the required verification has been completed. If the process encounters an error such as a required field being missing, column names that have changed, or a value being out of range, the data do not progress to the next step. Once a failure is detected, a notification is sent to alert about the failure. The notification allows researchers to examine the data at the point of failure and either obtain error-free data or update the validation schema to match the changes. The system uses individual parquet files instead of a database warehouse or data ecosystem; the process could be considered a combination of the common data processing approaches of extract, transform, and load (ETL) and extract, load, and transform (ELT) as we preserve and present both the extracted data along with the final transformed data.

**Figure 1:**
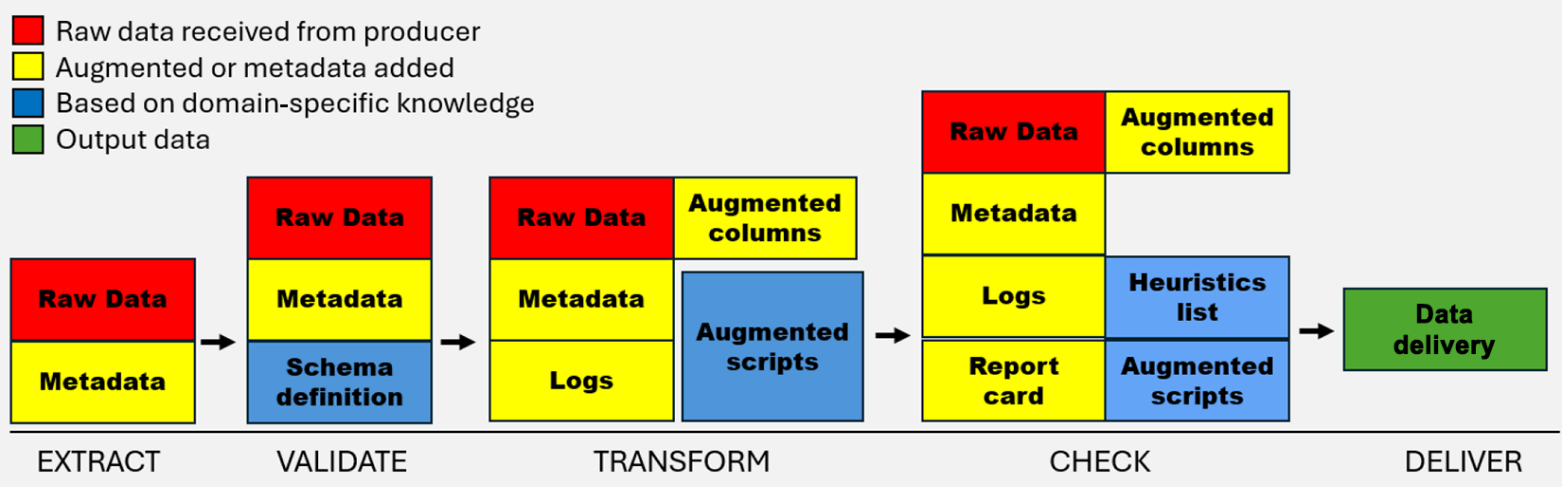
Illustration of the steps for automation of data integration implemented in the Purdue Animal Science Data Ecosystem (PASDE).

In the extraction stage, raw data are fetched or received from disparate sources. Methods include but are not limited to API calls, retrieving files from remote storage systems, and database queries. This process involves establishing connections to the data sources using appropriate protocols and technologies, such as RESTful API for web services or Globus file transfer. This step sometimes tracks previously extracted data to obtain only the data that have changed or are new. Data are collected from automatically generated reports (e.g., parlor management, farm management, feed management systems, automated milking systems), accessed via an API (e.g., weather) or sent from a third-party provider periodically (e.g., genetic evaluation systems). For generated reports, Globus (Chicago, IL) is used to transfer data from the site of collection to the centralized data ecosystem, automating the data pipeline. Ingestion of the source data files as-is with very limited transformation was used and the output was parquet files on a shared file system.

1 Validation: This stage ensures that the data match the predefined raw data schema definition. This step should perform a limited schema validation. It ensures that the data types, formats, and data quantity are acceptable to maintain minimum data quality, structural consistency, and integrity of the data. This step is still not domain-level or heuristic verification of the data. The initial validation step ensures that data fields are matching metadata specifications. Validation scripts are run with Python and may be available upon request (https://github.itap.purdue.edu/PASDE/PASDE-Pipeline-Notebook).
2 Transformation: In this stage, the data are augmented to conform to a predefined structure and prepared for final downstream consumption, analysis, and storage. This stage involves cleaning, filtering, enriching, aggregating, and formatting data according to the user requirements and analytical needs. Examples would be standardizing dates, converting imperial units to metric units, and joining datasets. These transformations are executed in Python to standardize units, dates, and other unifier variables among users.
3 The check stage is an additional component of validation in which incoming data are thoroughly compared with predefined domain-level heuristics to ensure adherence to the expected values, format, structure, and quality standards. This stage plays a crucial role in maintaining data integrity, identifying potential issues, and providing better insights into the quality of the data to be delivered. This process typically involves generating logs, checking against a heuristic list based on metadata, and producing a report card summarizing the results.
4 Delivery is the final stage after completing all prior steps, this stage delivers or packages the reference data to its final format in Apache Parquet and places it in the PASDE data ecosystem. For end users' access, Jupyter Notebook provides a server-based solution for data exploration and manipulation without overwhelming local system resources. These notebooks are an effective way to document data analysis workflows and share them with other users.

During the process of data integration (formation of a data ecosystem; Figure 2), there are many challenges to overcome. Common issues observed in the development of PASDE and the solutions proposed by our team to minimize their effects are described next. First, there is a need for a common ID. Data generated on individual animal basis require identification of animals paired with a measurement or attribute. A way to combat data interoperability issues when systems do not use a common identification system is the development of data mapping to show how individual variables can be connected. Although systems may have their own identification systems, they often report information such as birthdate and farm identification number, which can be used to connect data across systems. A clear understanding of variable definitions through comprehensive metadata generation is also used to correctly connect variables with the same meaning even though they may have different names across programs. A goal for creating metadata is to enforce a specific schema onto data entering the data ecosystem to improve usability (Oram, 2015).

**Figure 2:**
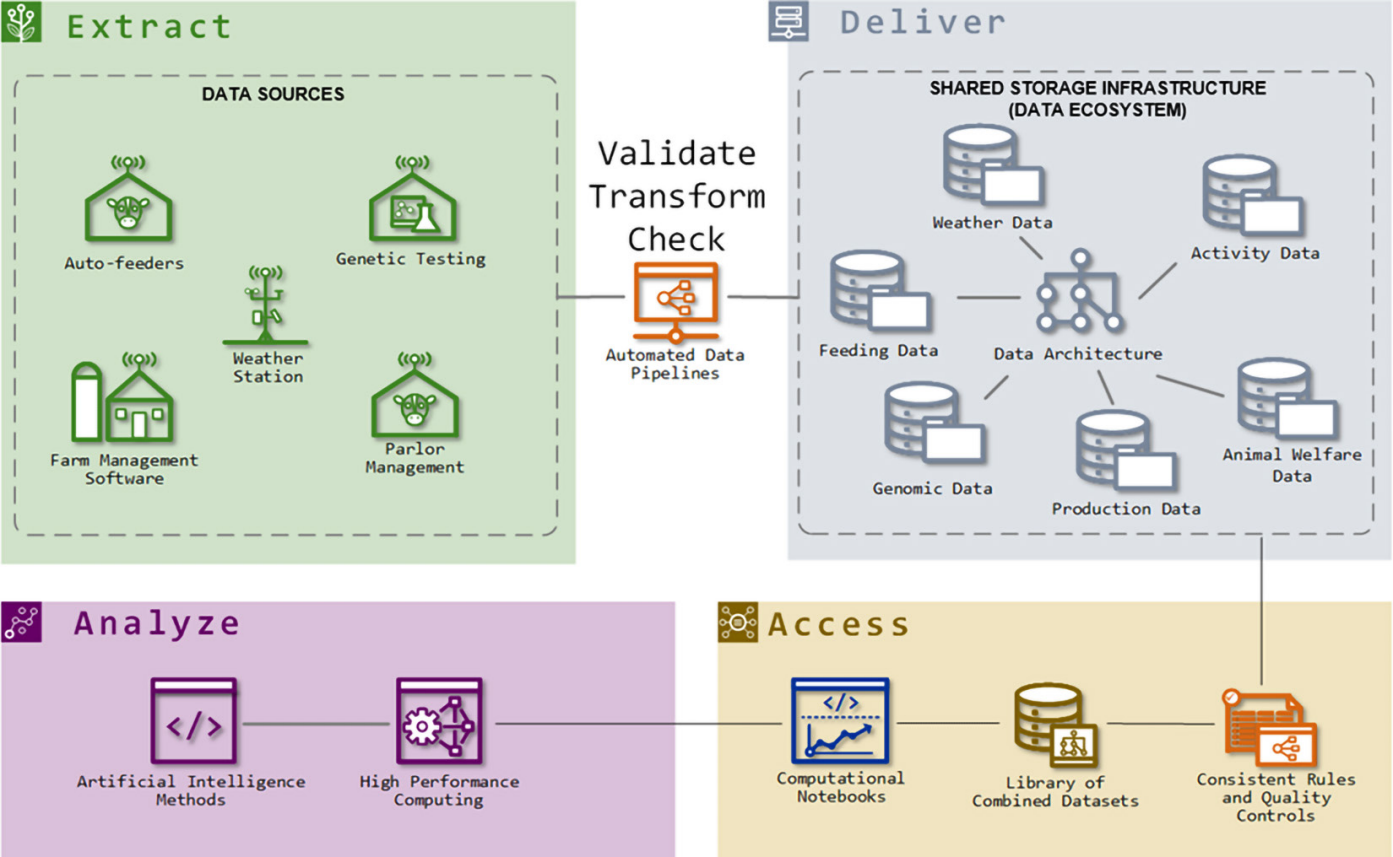
Description of the data pipeline implemented in the Purdue Animal Science Data Ecosystem (PASDE).

Examples of challenges within datasets that were identified through metadata generation are described here:

1 Date: To temporally connect data across time, date fields are essential. Across and within software programs used, date fields were not consistently formatted. For example, our preferred date format was YYYY/MM/DD; however, multiple formats were received. All date fields were converted to a common format during the derived file format generation. All data were ingested as original datasets and then a common date format was applied to generate derived datasets with consistent date formats.
2 Null data: Individual sensors and software represent null data in a variety of ways (i.e., -, ., 0, N/A [N/A = not available], –, or blank). The meaning of these null values may indicate that there is a missing datapoint or measurement that was not collected; however, a “0” may be interpreted differently based on the system or data source. Therefore, data tidying in the conversion between original and derived datasets standardizes null data, which have different rules based on data sources.

Some important steps to be considered when developing a data ecosystem consists of the creation of detailed and up-to-date metadata files (available from the authors upon request). Metadata files for data integration are critical because they provide the necessary context to correctly interpret, integrate, and use the data. Some key components of metadata are as follows: (1) Data identification and description, including data source, title or name of the dataset, data description, and version information (e.g., equipment, software, algorithms). (2) Data provider information, including data owner or custodian, date of data download, and source of data. (3) Structural information, which consists on a description of the data structure. This component should describe the dataset field names. Data type (e.g., integer, string, date), data format (e.g., YYYY/MM/DD for dates), units of measurement, and examples of values. (4) Data quality and accuracy, including information about data completeness, data accuracy (e.g., validation steps performed), data update frequency, and data processing history. (5) Technical information, including file format (e.g., .csv, JSON), data storage location, and access protocols (how to access the data). (6) Integration-specific information such as mapping rules or correspondences between fields, transformation logic (e.g., renaming fields, changing units), and dependences (on other datasets or variables). Detailed metadata are essential for data standardization and optimal usage of sensor-based datasets. In this context, there is a need for institutional collaborations to establish repeatable and scalable dairy data projects. International organizations such as the International Committee on Animal Recording in partnership with universities and other research organizations are expected to play a key role in the development of standards and guidelines for metadata development and use of sensor data. Furthermore, there are other relevant points to be considered such as data ownership, data exchange agreements, and privacy (Lokhorst et al., 2019).

Data quality control is a major step in ensuring high-quality data are used for research and development of application and decision-making tools. Implementing automated pipelines for data quality control facilitates the detection of errors, maintenance of data integrity, and generation of more accurate end results. It is worth noting that quality control may be specific to the data analytics purpose (e.g., management or genetic selection). These pipelines should perform data profiling to detect patterns and anomalies in the data. This can be based on descriptive statistics of the data (e.g., minimum and maximum values, average, and SD) and generation of plots. In this step, one should also implement validation checks to verify formats of data points and range values based on information provided in the metadata and remove duplicated values. After identifying data quality issues and cleaning the data, one could perform data imputation as is commonly done for genomic data (i.e., genotype imputation), remove outliers, and standardize data formats across all data sources that will be integrated. As new datasets are added or the existing data schema changes, the underlying configuration files in YAML (https://yaml.org/) format are easily updated. The YAML format makes the configuration files both human and machine readable.

The datasets integrated through PASDE have been used for a wide range of purposes. Examples of using these integrated datasets include evaluating biological and environmental factors that affect animal performance (e.g., Hurst et al., 2022; Montes et al., 2023) and defining novel traits for breeding purposes and the investigation of their genetic background, including milkability traits from milking robot data (e.g., Pedrosa et al., 2023), dairy cattle resilience based on variability in daily milk yield (e.g., Chen et al., 2023), calf resilience based on variability in milk consumption (e.g., Graham et al., 2024), and dairy cow behavior (e.g., Pedrosa et al., 2024). Although other tools with similar goals have been developed, there is a lack of documentation on the pipelines and procedures used as done in this technical note.

In summary, the developed PASDE data ecosystem has enabled the integration of various data sources, which were subsequently used for research projects in different disciplines. This data infrastructure will continue to be developed as additional data sources and new or updated computing tools become available.
